# Understanding the Preferences and Considerations of the Public Towards Risk‐Stratified Screening for Colorectal Cancer: Insights From Think‐Aloud Interviews Based on a Discrete Choice Experiment

**DOI:** 10.1111/hex.14153

**Published:** 2024-07-19

**Authors:** Rebecca A. Dennison, Reanna J. Clune, Stephen Morris, Chloe Thomas, Juliet A. Usher‐Smith

**Affiliations:** ^1^ Primary Care Unit, Department of Public Health and Primary Care University of Cambridge Cambridge UK; ^2^ Sheffield Centre for Health and Related Research, School of Medicine and Population Health University of Sheffield Sheffield UK

**Keywords:** acceptability, cancer screening, discrete choice experiment, health policy, qualitative interview, risk stratification

## Abstract

**Context:**

Risk stratification has been suggested as a strategy for improving cancer screening. Any changes to existing programmes must be acceptable to the public.

**Objective:**

This study aimed to explore the preferences and considerations of individuals relating to the introduction of different risk‐based strategies to determine eligibility for colorectal cancer (CRC) screening.

**Study Design:**

Participants completed a discrete choice experiment (DCE) within online interviews. Nine conjoint‐analysis tasks were created, each with two potential CRC screening programmes. The attributes included personal risk of CRC, screening invitation strategy and impact. Participants chose between programmes while thinking aloud and sharing their thoughts. Transcripts were analysed using codebook thematic analysis.

**Participants:**

Twenty participants based in England aged 40–79 years without previous cancer history or medical expertise.

**Results:**

When choosing between programmes, participants first and primarily looked to prioritise saving lives. The harms associated with screening were viewed as a surprise but also felt by most to be inevitable; the benefits frequently outweighed, therefore, harms were considered less important. Risk stratification using individual characteristics was considered a nuanced approach to healthcare, which tended to be preferred over the age‐alone model. Detailed personal risk information could be taken more seriously than non‐personalised information to motivate behaviour change. Although it had minimal impact on decision‐making, not diverting resources for screening from elsewhere was valued. Individuals who chose not to provide health information were considered irresponsible, while it was important that those with no information to provide should not lose out.

**Conclusion:**

Risk‐stratified CRC screening is generally aligned with public preferences, with decisions between possible stratification strategies dominated by saving lives. Even if attributes including risk factors, risk stratification strategy and risk communication contributed less to the overall decision to select certain programmes, some levels more clearly fulfilled public values; therefore, all these factors should be taken into consideration when redesigning and communicating CRC screening programmes.

**Patient or Public Contribution:**

The primary data source for this study is interviews with 20 members of the public (current, past or future CRC screening invitees). Two public representatives contributed to planning this study, particularly the DCE.

## 1 Introduction

Personalised or risk‐based approaches have been proposed as strategies to improve colorectal cancer (CRC) population screening programmes in the United Kingdom and many other countries [1, 2]. Specifically, such programmes have the potential to increase cost‐effectiveness and appropriateness of colonoscopy allocation, improve CRC incidence and outcomes and reduce the risk of psychological or physical harms associated with the screening pathway (since the risk of bleeding, bowel perforation and death are rare but clinically important at an estimated rate of 24, 6 and 0.3 per 10,000 colonoscopies, respectively) [2, 3, 4, 5].

In risk‐stratified screening, instead of following a uniform schedule, individuals undergo a cancer risk assessment, and their screening programme is adapted according to their risk of having or developing cancer. Risk stratification could be incorporated into CRC screening in multiple ways at different points on the screening pathway and/or using different algorithms to estimate cancer risk [3, 6]. The risk assessments could include demographics such as age and sex, lifestyle factors such as diet and physical activity, family history of CRC or genetic risks [7]. Introducing risk stratification at the point of determining screening eligibility—where people would be invited for their first screening test at a younger age if they have a higher risk of CRC and vice versa—has been modelled to improve cancer outcomes and be cost‐effective compared to the current programme in England: using a comparable total number of screening tests; it could result in 218 fewer cases of CRC and 156 fewer CRC deaths per 100,000 people [5].

Consensus on the best way to implement risk stratification into CRC screening programmes has yet to be reached [8]. In addition to considerations of infrastructure, coordination and integration, benefits and harms, value for money and quality and performance management, an essential principle that a screening programme must meet is acceptability and ethicality [9]. Previous research has shown that, despite some concerns, risk‐stratified screening tends to be acceptable to the public and healthcare professionals [10, 11, 12]. In the United Kingdom, members of the public supported the concept of risk‐stratified CRC screening and valued the benefits that could result when considering the concept from a societal perspective [6]. Nonetheless, they were concerned about the implications for people with a low estimated risk of cancer who would be invited to screening at an older age than others and the potential for (apparent) inequalities in screening access [6], concepts that have been observed and discussed more widely [3, 11, 13, 14, 15, 16]. This serves to illustrate how important it is to carefully consider the public's perspective in the design of screening programmes to maintain their trust in the healthcare system and engagement with screening.

In a recent discrete choice experiment (DCE), we explored individual‐level views by asking members of the public to select their preferred potential risk‐stratified CRC screening programmes [17]. We found that they prioritised the number of CRC deaths prevented by screening, followed by the number of people experiencing screening harms. Attributes associated with personal risk of CRC had a small influence while, perhaps unexpectedly, the screening strategy itself was least important. This present study aimed to elicit participants' reasoning behind these decisions and how they went about the process. This allowed further, in‐depth scrutiny of the preferences of individuals in England concerning the introduction of different risk‐based strategies to determine eligibility for CRC screening through the completion of the same survey as part of a qualitative interview.

## 2 Methods

### 2.1 Study Design

Participants completed an online DCE about the introduction of risk stratification into CRC screening eligibility while thinking aloud. By asking participants to verbalise their thoughts and spontaneously report what goes through their minds while answering the questions, this approach provided rich data on cognitive processes.

### 2.2 Survey Design

The design and development of the DCE have been detailed elsewhere [17]. Briefly, a series of conjoint‐analysis tasks were created to assess the impact of features that had previously been identified as important to the public on participants' choices: risk of CRC, participants invited for cancer screening and the impact of the screening programme (as described in Table 1). The levels chosen were based on data from the MiMiC‐Bowel model [5, 18], and to reflect a plausible and clinically relevant range while also avoiding extreme values.

**Table 1:** Summary of attributes and levels used in the DCE.

Attribute	Definition and possible levels
Personal risk of CRC	
Risk factors	Individual characteristics and CRC risk factors collected from each person Age Age and sex Age, sex and lifestyle risk factors Age, sex, lifestyle and genetic risk factors
Feedback level	Level of feedback provided on individual risk of CRC Generic feedback (information about CRC risk factors plus prevention advice, but no personalisation) Basic personalised feedback (information about CRC risk factors, prevention advice and whether they had a high, average or low risk of CRC) Detailed personalised feedback (information about CRC risk factors, prevention advice and what constituted their high, average or low risk of CRC)
Who is invited for screening	
Screening strategy	When people will be invited to start screening (including a diagram) All at the same age High‐risk invited earlier (people at high risk will be invited before people at average and low risk) Risk‐stratified (people at high risk will be invited before average risk, and low risk after average risk)
Resource use	Resources needed for screening Same as the current CRC screening programme More than the current CRC screening programme
Default risk	How to handle people with no information with which to calculate the risk of CRC Treat them as low risk when inviting them to screening Treat them as average risk when inviting them to screening Treat them as high risk when inviting them to screening
Impact of the screening programme	
The number of deaths prevented	Number of deaths from CRC that will be prevented by screening, per 100,000 people 300 700 850 1300
Number of people harmed by screening	Number of people who will experience physical harm from screening (bleeding, damage to the bowel or death), per 100,000 people 2 20 60 100

*Note:* See the survey outline for full descriptions of the attributes and levels [17]. Nonsense/illogical combinations were excluded.

**Figure 1:**
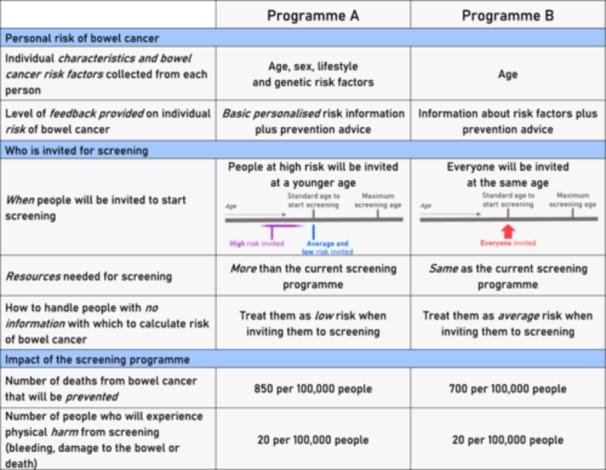
Example of a choice question.

All participants were presented with nine questions. Each question included two programmes and participants were asked to choose between them in a forced‐choice elimination format. An opt‐out (no screening) option was not available. An example of one conjoint‐analysis task is given in Figure 1.

Before beginning the conjoint‐analysis tasks, participants were provided with an explanation of the purpose of the DCE and each attribute and level. We sought to provide sufficient information to aid understanding so that participants could take each attribute into consideration when completing the DCE but not burden them with too much reading. The information was followed by a series of true or false questions relating to the attributes to facilitate understanding. Once participants had completed the conjoint‐analysis tasks, they were asked to rate how easy or difficult they found the tasks and to rank the different attributes in order of importance to them. Finally, participants provided demographic information regarding age, sex, ethnicity, socioeconomic status, education level and family history of cancer using validated measures.

### 2.3 Participants and Recruitment

Participants were recruited via a market research company (iPoint Research Ltd, Middlesex, UK) and purposefully stratified by age, sex and educational background. Participants aged between 40 and 79 were invited to take part, while individuals with a personal history of cancer, expertise in medicine or who had previously taken part in similar studies conducted by these researchers were excluded.

Participants were recruited using a standard template and provided with the participant information sheet before deciding to participate. Informed written consent was obtained in advance and then confirmed verbally at the start of the interview, with the option of withdrawing from the study clearly explained. Participants were encouraged to take part on a computer or tablet and in a quiet, private location. They were reimbursed by the recruitment company at their recommended rate. They had no direct contact with the research team before taking part.

### 2.4 Data Collection

Data were collected during face‐to‐face, hour‐long online interviews by the first author, a female postdoctoral researcher with qualitative research experience. Interviews were conducted online using Zoom videoconferencing software (California, USA) and the survey was hosted on the Gorilla Experiment Builder (https://www.gorilla.sc; Cambridge, UK). At the start of the interview, the researcher introduced themself, explained the interview process and asked the participant to practice thinking aloud. Once this was understood, they then began the online survey using screensharing. After the participant read each section of background information about CRC screening and its attributes, the researcher asked for their initial thoughts and feelings about the information and clarified any misunderstandings where appropriate. For the DCE questions, participants were asked questions like why they selected the screening programme given each of the attributes and their levels. Participants discussed the elements of each screening programme that appealed and did not appeal to them and explained any trade‐offs that they made between programmes. The interviews were recorded using Zoom and transcribed verbatim by an external company.

### 2.5 Analysis

Codebook thematic analysis was used to synthesise the findings [19]. The researchers first familiarised themselves with the data by immersing themselves in the transcripts, engaging in reflections and conducting iterative cycles of reading to inform the development of a list of codes that reflected the attributes included in the DCE. The first two authors coded the transcripts using NVivo 12 software (Lumivero, Colorado, USA). Coding was reviewed by creating a summary of all the codes on post‐it notes and grouping them based on their similarities to inform potential themes within each attribute. These themes were expounded, and examples were identified. Additionally, summary statistics of participant characteristics and ranking of the attributes were calculated, and, without formal statistical testing, compared to those reported in the quantitative survey findings [17].

## 3 Results

### 3.1 Participants

A total of 20 participants were interviewed in September 2022. Participants with a range of characteristics were included (Table 2 and Supporting Information S1: Table 1). A balanced representation of males and females took part. Most participants were middle class (90%) and had a degree‐level education (60%). Participants' beliefs about cancer, worry about cancer and views on screening are shown in Supporting Information S1: Table 2.

**Table 2:** Key participant demographics.

	*N* (%)
Total *N*	20 (100)
Age range (years)	
40–49	7 (35)
50–59	6 (30)
60–69	2 (10)
70–79	3 (15)
Sex	
Female	10 (50)
Male	10 (50)
Ethnicity	
Asian or Asian British	2 (10)
Black or African or Caribbean or Black British	3 (15)
Mixed or multiple ethnicity	1 (5)
White	14 (70)
Education	
Completed A levels or equivalent, or less education	4 (20)
Completed further education but not a degree	4 (20)
Completed a bachelor's degree	9 (45)
Completed a master's degree or PhD	3 (15)
Social grade	
Middle class	18 (90)
Working class	2 (10)
Cigarette or cigar smoking status	
Never smoked	8 (40)
Used to smoke	10 (50)
Smoke up to 20 cigarettes or cigars per day	2 (10)
Family history of cancer	
Yes	5 (25)
No	15 (75)

### 3.2 Ranking of Attributes of CRC Screening Programmes

**Figure 2:**
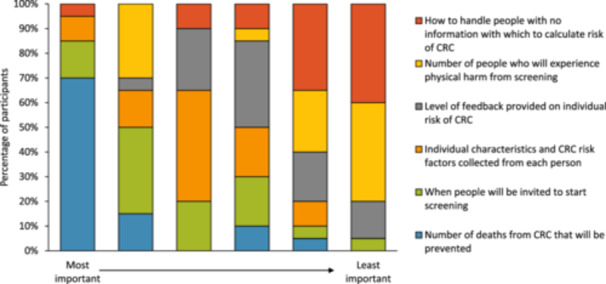
Participants' ranking of attributes to inform eligibility for CRC screening programmes (*n* = 20).

After completing the DCE, the participants ranked the attributes as reported in Figure 2. Their priorities were largely consistent with those of the population survey participants (*n* = 1132 with valid ranking data) [17], although survey participants ranked seven attributes whereas interview participants ranked six because resource use was not presented as a separate attribute in the ranking question in this study. The number of CRC deaths prevented was one of the two most important attributes for 17 (85.0%) interview participants, as it was for 1038 (91.7%) survey participants. The other attribute relating to screening programme outcomes, physical harms from screening, was ranked as important to some interview participants (six participants, 30.0%) and unimportant to others (13 participants, 65.0%). Survey participants tended to think that this attribute was more important than the interview participants did, with 556 (49.1%) ranking it as one of the top two most important attributes compared to 254 (22.4%) ranking it as one of the two least important attributes. How to handle people with no information with which to calculate CRC risk was least important, with 15 (75.0%) interviews and 789 (69.7%) survey participants ranking it last or second last.

Thinking aloud whilst completing the DCE, participants explained how each attribute contributed to their decision between programmes, their preferences for certain levels, as well as additional considerations relating to each attribute. These are described in the order in which participants collectively ranked the attributes and are summarised in Table 3. Additional supporting quotations are included in Table 4. CRC risk factors and screening strategies are presented together as participants' thoughts on these attributes are interlinked.

**Table 3:** Summary of participants' considerations in relation to each attribute and level.

Attribute	Preferences	Rationale and other key points
Personal risk of bowel cancer		
Risk factors	Include multiple risk factors	Including more CRC risk factors was nuanced and individualised In comparison, sex‐ and/or age‐based screening was illogical
Feedback level	Provide detailed feedback on personal CRC risk	Detailed feedback could motivate prevention behaviours Detailed feedback could also induce anxiety about CRC risk Those at low risk could develop a false sense of security
Who is invited for screening		
Screening strategy	Invite people to screening according to their CRC risk	Both age‐based and risk‐stratified screening fitted with participants' value systems, in different ways Risk‐stratified screening was intuitive and could enable younger people to access screening
Resource use	Minimise resource use	Using the same or fewer resources for screening was preferred to reduce the burden on the NHS Weighing resource implications with lives saved was distasteful
Default risk	Vary according to the reason for no risk level being available	Individuals who choose not to engage should not start screening early Individuals who cannot provide risk information should be treated as average or high risk Screening as average risk could be considered a compromise
Impact of the screening programme		
The number of deaths prevented	Prevent more CRC deaths	Number of lives saved was an objective measurement of the effectiveness As the aim of screening, it was emotive and hard to select against
Number of people harmed by screening	Minimise CRC screening harms	Physical colonoscopy harms surprised many participants The magnitude of harm was interpreted as low Screening harms were an acceptable risk when weighed against the potential benefits

**Table 4:** Supporting quotations.

Number	Quotation and participant characteristics
Prioritise the number of lives saved from CRC	
1	Because that is really the point to prevent death, isn't it, of the programme? (P13; 55 years, female, White ethnicity, degree education)
2	I think the first thing I seem to look at is the number of deaths prevented, because that seems to be like the most sort of factual way of working it out … it's almost like I just feel like why look at the other stuff because it seems to make sense to go for the one that will obviously save them the most people. (P14; 52 years, female, White ethnicity, degree education)
3	I don't really like either of them but I think just the human element I think I'm going to go with B because more people are saved. (P03; 45 years, female, Black ethnicity, no degree education)
Use a variety of individual characteristics to inform when people will be invited to attend the screening	
4	So my first thought is like, ‘Oh yes, definitely the high‐risk people should go first,’ sort of thing, that they should be tested earlier at a younger age, and then I'm thinking of … well, [it's a] really difficult decision, isn't it, because it's fair to ask everyone at the same time. (P15; 46 years, female, White ethnicity, degree education)
5	Age and sex doesn't feel like enough to be giving the NHS an idea of who needs to be tested, I think I will go with A just because B isn't really enough. (P16; 51 years, female, White ethnicity, degree education)
6	Because everyone will be invited according to their risk, you know, and whereas, yeah I'm going to go with Programme B, that's so obvious. And they would be invited at a younger age if they're at high risk, which is, they should be. (P10; 78 years, female, White ethnicity, no degree education)
7	I feel like if we do know about factors, then we ought to include them all that we know about now. It just feels perhaps we'd be ignoring information that we could use. So I think that's the way I would do it, yes. (P15; 46 years, female, White ethnicity, degree education)
8	So, if I'm right, that age and sex would be older men because they're more likely to have it and we're more likely to save more of them because we've screened them […] Where's the equality in that, aye? It's maths again, isn't it, those figures? (P05; age not recorded, male, Black ethnicity, no degree education)
9	… you're working from the point that whatever resources you have are finite, limited. And if you put resources into a specific project where's that money going to come from? Will it be taken out of screening of other illnesses? (P04; 53 years, male, Asian ethnicity, degree education)
10	If you know nothing about your sample of 100,000, is it sensible to treat them all as high risk? […] I don't want to get political, but you have to treat them with the reality of the world which is that the NHS is massively underfunded. (P17; 57 years, male, White ethnicity, no degree education)
Provide detailed information on the personal risk of CRC	
11	You'd be more shaken up I think, because obviously you could hear the general advice, but you might think, oh it doesn't affect me, but if they said, ‘Actually, you are at greater risk’, I think it would sort of scare you into then making some changes, probably. (P14; 52 years, female, White ethnicity, degree education)
Reduce the number of people who experience screening harms	
12	So a number of people will experience physical harm from screening. Wow. Oh, surgery! Death! Wow, okay, that wouldn't really encourage me … (P17; 57 years, male, White ethnicity, no degree education)
13	When it's only 20 out of 100,000 people, if there's only 20 that are going to experience the harm, the good that it will do will certainly outweigh the 20 people that might find harm from it. I know that sounds a bit harsh. No‐one wants 20 people to be harmed, but it's definitely worth the risk. (P18; 49 years, female, White ethnicity, no degree education)
14	If you take the emotion out and look at it with the cold light of numbers then, I don't like to use the phrase, acceptable risk, but it would appear to be […] that's the only way I would look at it I think. (P04; 53 years, male, Asian ethnicity, degree education)
15	I don't like that term [physical harm] but yes, [if you] can reduce the risk to anybody, that's got to be a good thing. (P07; 52 years, male, White ethnicity, no degree education)
Determine default strategies according to the reason why no risk information is available	
16	They've taken a personal decision … yeah, I was going to say it's their own fault if then that didn't work out for them further down the line. I don't mean it in that brutal term but … (P06; 41 years, male, White ethnicity, degree education)
17	I think handling people with no information as low risk is a bit unfair […] because there's a subset really of people who just do not have the information. (P15; 46 years, female, White ethnicity, degree education)

### 3.3 Prioritise Number of Lives Saved From CRC

Participants typically sought out the number of deaths prevented before examining all other attributes of the screening programmes since this was the most important and defining factor in their decision‐making. Even in contexts where participants did not particularly like either of the two programmes presented to them, their decision was driven by the lives saved. Reasons given for this included that it was an obvious, objective and logical measurement of a CRC screening programme's effectiveness (Quotes 1 and 2). The number of deaths prevented also served as a ‘yardstick’ for effectiveness when comparing programmes (P01; 70 years, male, White ethnicity, degree education), as participants felt that it was easier to evaluate programmes using a tangible outcome such as this.

Considering the number of lives saved also evoked emotional responses from participants who expressed a moral obligation and responsibility to select programmes that had the most benefit for others (Quote 3). Choosing programmes that saved the most lives was deemed as the most compassionate approach. This moral obligation overrode hesitations they had regarding personal attitudes towards risk stratification and concerns for resource allocation, with the view that higher levels of resource use were an acceptable trade‐off. Similarly, trade‐offs against lives saved from CRC were infrequent.

### 3.4 Use a Variety of Individual Characteristics to Inform When People Will Be Invited to Attend Screening

Participants considered the pros and cons of both screening everyone at the same age and the two options for risk‐stratified screening. They frequently looked into the factors used to estimate risk when considering when people would be invited to attend screening; therefore, views on these attributes have been assimilated. The impact of these attributes on the decision between programmes was also considered in the context of the number of lives saved through each strategy.

To some participants, it was considered fair to invite everyone at a set age for screening. Others were favourable towards this approach because it had the best fit with their belief that healthcare should be available to all. Some also stated preferences for anybody to be able to access screening whenever they wanted it. On the other hand, other participants expressed concerns about age or age and sex‐based screening, describing it as ‘quite blunt’ (P02; 65 years, male, mixed ethnicity, degree education) and that ‘I don't think inviting everyone at the same age would work because there are people who are at higher risk‘ (P03; 45 years, female, Black ethnicity, no degree education) (Quotes 4 and 5).

Conversely, risk‐stratified screening was considered sophisticated, and it was logical that inviting people with a higher risk of CRC at a younger age would lead to earlier diagnoses (Quote 6). One participant said that their ‘intuitive response is where you can help the most that's where you should start’ (P04; 53 years, male, Asian ethnicity, degree education). The main consideration regarding people at lower risk being invited at an older age was that they would lose out on screening, which might have negative outcomes. Others felt that people were more likely to lose out if age‐based screening continued because risk stratification would give people at higher risk the opportunity to be screened at a younger age.

Risk‐stratified screening was only presented to participants where risk was based on multiple factors. Within fully risk‐stratified and high‐risk invited earlier strategies, participants showed preferences for screening programmes that used a variety of individual characteristics over programmes that used fewer. They recognised that age, sex, lifestyle and genetics all contribute to the risk of CRC; therefore, using them within a risk assessment to inform screening made sense (Quote 7). Several participants anticipated further benefits such as the screening invitation carrying more gravity. A few participants raised some concerns about providing risk information; for example, how data would be stored, protected and kept up to date, and that some people may find it ‘complicated’ or ‘overwhelming’ (P05; age not recorded, male, Black ethnicity, no degree education). One participant suggested that those at the highest risk might be less likely to provide the information as they would fear that their high‐risk status would be confirmed. Others suggested that they would be happy to provide the necessary information or a sample for genetic testing if they were asked to. These points tended to be reflections that did not feed into their decision.

Furthermore, a couple of participants acknowledged that risk stratification could differentially impact subgroups of society, although they did not conclude what impact this had on programme preferences. For example, the potential for men to benefit more than women or older people more than younger people (Quote 8).

### 3.5 Use Minimal Resources

The use of resources did not clearly or often sway participants' decision‐making since it was outweighed by other attributes. If more resources would be required for their preferred programme, it was described as ‘a bit of a reservation’ rather than causing them to decide against it (P06; 41 years, male, White ethnicity, degree education).

Nonetheless, the participants would not want to increase, or in some cases even wanted to reduce, the burden on the NHS if risk‐stratified screening was introduced. They were quick to acknowledge that if spending on CRC screening increased, it would require further investment or funds to be diverted from other services, which was unfavourable (Quotes 9 and 10). Furthermore, they related resource implications to other aspects of risk‐stratified screening. For example, they considered the potential for conducting risk assessments, different screening strategies and defaulting people with no risk information as high risk to result in increased NHS workload. Conversely, just one participant stated that ‘is it more that the public should be getting what they're worth […] spend some more money’ (P05; age not recorded, male, Black ethnicity, no degree education).

Although they acknowledged that it was necessary, notably several participants found considering the resource implications uncomfortable or distasteful. Specifically, ‘it's that horrible decision that someone has to make about that cost effectiveness to save how many lives’ (P07; 52 years, male, White ethnicity, no degree education) and ‘essentially in the NHS they're dealing with people's lives; that's the currency’ (P06; 41 years, male, White ethnicity, degree education). Many therefore also suggested their preferences between programmes in the context of the ‘ideal world’ where resource restraints would not be a consideration, as noted in Quote 10.

### 3.6 Provide Detailed Information on Personal Risk of CRC

The strong preference for detailed feedback on their risk of CRC was based on the view that ‘targeted’ and ‘bespoke’ feedback had the potential to lead to positive outcomes such as increased screening compliance and motivation for behaviour change (P06; 41 years, male, White ethnicity, degree education). This was particularly focused on those at higher risk; some noted that more basic feedback would be sufficient for those at average and/or low risk. Participants thought that it would give those at higher risk an opportunity to evaluate their lifestyle and prioritise areas for improvement (Quote 11), while those at low risk would have the tools to maintain their low risk. This would be ‘part of a cycle of education’ that leads to prevention (P04; 53 years, male, Asian ethnicity, degree education). The one exception that some considered was the provision of detailed feedback on genetic risk, whereby its fixed nature was felt to potentially result in participants feeling burdened by a high‐risk score. Generic information about CRC risk factors, by comparison, was not considered useful, and could even be risky as it may not be received with the appropriate level of gravity and so result in individuals with a higher risk not taking adequate care.

The participants did, however, give careful regard to the potential for detailed feedback on individual risk to result in anxiety and worry or a false sense of security for those categorised as lower risk, as ‘if I'm coming out as low risk maybe I ignore that symptom that might be the start of something more sinister’ or not attend for routine screening as they feel it may no longer be necessary (P06; 41 years, male, White ethnicity, degree education).

### 3.7 Reduce the Number of People Who Experience Screening Harms

In the wider discussions, particularly when reading the information at the start of the study, participants responded to the idea of screening harms in different ways. Several participants expressed shock and concern when hearing of the potential physical harm associated with the CRC screening pathway as a whole. Screening had been viewed as ‘virtually risk free’ (P04; 53 years, male, Asian ethnicity, degree education), which was because some had only considered the faecal immunochemical test (FIT) while others had assumed that a common procedure like colonoscopy would be ‘foolproof, nobody would experience any kind of harm or adverse effect’ (P04; 53 years, male, Asian ethnicity, degree education) and Quote 12. For these individuals, the potential harms associated with colonoscopy were deemed as ‘quite off‐putting’ (P08; 41 years, male, White ethnicity, degree education), and left them feeling ‘more worried than convinced’ (P05; age not recorded, male, Black ethnicity, no degree education). On the other hand, another view was that harm was inevitable, as ‘there is a risk to everything that you have done’ (P09; 67 years, male, White ethnicity, no degree education).

Whichever view they took regarding harms, most participants viewed the likelihood of these harms as low enough that the risk was acceptable and therefore placed screening harms at lower priority when ranking the attributes of screening programmes (Quote 13). Although the participants who ranked this attribute more highly considered that the harms within the range presented were still ‘far too high […] a hell of a number’ (P08; 41 years, male, White ethnicity, degree education), those with the opposite perspective described them as ‘almost non‐existent it's so low’, ‘so slight’ (P09; 67 years, male, White ethnicity, no degree education] and ‘too negligible’ (P10; 78 years, female, White ethnicity, no degree education). As a result, these latter participants appeared to be able to disconnect emotionally from the individuals who may experience these harms, and instead consider only the benefits as those would always outweigh the potential harms (Quote 14).

Regardless of its contribution to decision‐making yet more salient to those who considered it important, participants wanted to minimise the number of individuals who experienced harm (Quote 15). They stated that they ‘wouldn't want to be the one that did experience physical harm’ (P11; 71 years, female, White ethnicity, degree education) and further acknowledged that programmes that fail to consider this ‘may have the best intentions to save lives but you might actually be making people's lives worse’ (P08; 41 years, male, White ethnicity, degree education)*.* Therefore, programmes that are most effective in saving lives yet also made efforts to reduce the number of harms were preferable over those that just increased the number of lives saved.

### 3.8 Determine Default Strategies According to the Reason Why No Risk Information Is Available

Although it was unimportant in the context of screening programme preferences, participants thought that individuals without CRC risk information should be handled fairly, both individually and within a wider society. They identified two subsets of individuals who should be treated differently: first, those who intentionally do not provide information, and then those with no information to provide. This contributed to a lack of consensus regarding preferred levels for this attribute.

Several participants claimed that they ‘don't quite understand why anybody would not want to provide the information’ about their health for a risk assessment (P09; 67 years, male, White ethnicity, no degree education), even viewing them as ‘foolish’ for not taking responsibility for their own health and national healthcare resources (P06; 41 years, male, White ethnicity, degree education). Treating this group as high‐ or average‐risk by default would, therefore, be unjust as participants felt that they should not be given special treatment or be prioritised over those who have complied (Quote 16). Instead, individuals who choose not to provide information should be treated as low risk, which might even motivate participation.

The participants considered why people may not be able to provide information for a risk assessment and identified individuals who could not access information regarding their family history (e.g., because they have no contact with their biological family) and others who do not have the opportunity or resources to attend medical appointments, which would impact a risk assessment based on data within medical records. Participants believed that it would be unreasonable to screen people with no information to provide at an older age (Quote 17).

Other participants, such as those who did not distinguish between the two groups, were uncertain regarding the best level of this attribute and suggested to ‘treat them the same as an average person because you haven't got information to make another decision from’ (P12; 49 years, female, White ethnicity, degree education).

## 4 Discussion

We have explored in detail the public's priorities for risk‐stratified CRC screening, including the thought processes and beliefs that underlie the decisions that members of the public make when evaluating and weighing up screening strategies. We also showed the importance of considering key aspects of screening programmes that appear to have little influence on the public's preferences between policies but are, nevertheless, important to the overall acceptability. We discussed the distinct perspectives towards CRC screening benefits and harms, the importance of fairness around healthcare resources and missing data and the interaction between CRC risk factors, risk feedback and the risk stratification strategy.

It has been shown that the public prefers screening programmes that have the greatest impact on cancer outcomes [20, 21]. Indeed, the number of deaths prevented formed nearly two‐thirds of the relative attribute importance in the quantitative survey using the same DCE [17]. In the present study, we found that the number of lives saved was not only the most important but also the first metric that participants looked to when assessing the options. In many cases, this was because participants described feeling morally unable to choose against saving more lives and obligated to select the option with the greatest number of deaths prevented even if the levels of the other attributes within the programme were unfavourable. Conversely, perspectives on CRC screening harms, considering the pathway as a whole and not FIT testing in isolation, were complex and varied between participants: they could be surprising or inevitable, too high or acceptably low. Many were able to emotionally detach from screening harms and often considered them an inevitable trade‐off after preventing deaths. This fits with previous findings that have shown that the public has a poor understanding of, underappreciate or are accepting of the potential harms of screening [22, 23, 24, 25]. The ranked importance of screening harms is somewhat at odds with the survey findings in which minimising screening harms was the second most important after saving lives [17]. The survey participants may not have considered the harms in the same detail as the interview participants as they did not think out loud, as has been suggested that thinking aloud may be associated with a different way of thinking [26, 27].

Aside from their overriding views on cancer outcomes, our participants considered the benefits of the suggested CRC screening programmes to society beyond the number of lives saved without explicit prompting. In particular, they appreciated the implications of screening on healthcare resources, preferring programmes that would not require resources to be diverted from other parts of the NHS unless more lives would be saved from CRC. This may go some way to explain why the strategy in which those with a higher CRC risk are invited to the screening at a younger age was disfavoured in the survey [17], although it was also noted that this attribute was difficult to consider due to the absence of any quantification or more specific framing (e.g., in terms of reallocation of resources within healthcare or other public services), and the lack of understanding of NHS commissioning.

When considering the default risk attribute, we saw that the interview participants distinguished between people who choose not to and who cannot engage in risk assessments. They wanted those unable to provide risk information to be treated fairly and not miss out on screening yet advocated for personal responsibility and felt that people who choose not to take part should not be screened at a younger age. This view is similar to that shown in a previous survey in which 59% of participants felt that people who do not attend cancer screening are irresponsible (*n* = 1895) [22], yet is difficult to reconcile with risk assessments being optional [6, 28]. The potential for distinct interpretations of this attribute, which lead to preferences for different levels, may explain why an overall preference for default risk was not observed in the quantitative analysis [17].

Our findings also suggest that the selection of risk factors within any risk assessment and overall strategy for risk stratification may be more important to the public than indicated in the quantitative survey. In that survey, risk factors contributed only 11.1% of relative attribute importance and the screening strategy 3.6% [17]. This present study suggests that participants consider these attributes as integrated, together holding more importance. Furthermore, inviting those with the greatest need for screening (i.e., risk‐stratified screening based on multiple risk factors) was intuitive and logical whereas age‐based screening better fulfilled the principle of universal healthcare. Feedback on risk prediction, which again is impacted by the risk factors used, was anticipated to inform behaviour change and increase screening compliance. As identified previously [29], information may not directly lead to behaviour change but contributes to a positive and empowered perspective towards CRC screening.

While these perspectives were given in the context of risk‐stratified eligibility for CRC screening, many are likely to apply to other points on the screening pathway that are being considered for risk stratification, including screening interval and threshold for colonoscopy referral, and across other cancer screening programmes [6]. Lessons have also been learnt more generally about public considerations regarding the interaction between screening benefits, harms, healthcare resources and ethicality. This comprehensive understanding is valuable due to the degree of nuance we have highlighted, and is pertinent given calls for agreement on CRC risk stratification strategies since the current lack of consensus has been identified as a barrier to implementation [8]. While most relevant to policymakers, these findings also provide insights that will be important to consider when developing strategies for communication of changes to screening programmes, such as framing outcomes in terms of public values (greater screening benefits, resource allocation, fairness, etc.).

Using DCE methodology in the context of an interview enabled us to elicit public views on realistic risk‐stratified screening programmes with outcomes based on the MiMiC‐Bowel model [5, 18]. Although it did not truly replicate policy decision‐making, this enabled us to understand public priorities and thought processes when faced with conflicts, rather than simply idealistic views. The DCE methodology in the underlying survey, however, while enabling quantification of the contribution of different components and the trade‐offs individuals are willing to make, sometimes introduced confusion, such as if the risk‐stratified strategy saved fewer lives than age‐based screening in a particular question. Perhaps because of this, some participants still assumed that risk stratification would result in better outcomes regardless of the numbers presented in the question. This emphasises how logical the participants found risk stratification but highlights the challenges with this approach. With the exception of social class, the participants had a variety of demographics, which suggests we examined views from a range of perspectives according to these easily measured characteristics. Likewise, it is inevitable that individuals' backgrounds and perspectives influenced their responses, yet these are likely to be typical of those within wider society since demographics, views on cancer and ranking of the attributes were comparable between the survey and interview participants. Conversely, people without fluent English language were not eligible and should be focused on in separate studies. Future research should also seek the views of those less likely to take up CRC screening because it is important that risk stratification does not worsen inequalities in uptake [30].

## 5 Conclusion

Risk‐stratified CRC screening generally aligns with public values. The public approach screening benefits and harms differently. The benefits were widely considered the most important and objective measure of the success of a screening programme, so communication must clearly present the increase in the number of CRC deaths prevented by screening. By contrast, while harms are still important to include, selling risk stratification based on their reduction alone is unlikely to gain support from many people. In addition, explicitly sharing information about expected impacts on resource use and how people with missing data will be treated, potentially even distinguishing between the cause of missing data, may be required. Using a variety of individual characteristics to inform when people will be invited to attend screening is inherently logical, and being able to understand their own risk of CRC would be important to many.

## Author Contributions

**Rebecca A. Dennison:** conceptualisation, investigation, methodology, formal analysis, writing–original draft preparation, writing–review and editing. **Reanna J. Clune:** formal analysis, writing–original draft preparation, writing–review and editing. **Stephen Morris:** resources, supervision, writing–review and editing. **Chloe Thomas:** resources, writing–review and editing. **Juliet A. Usher‐Smith:** conceptualisation, funding acquisition, methodology, supervision, writing–review and editing.

## Ethics Statement

Ethical approval was obtained from the University of Cambridge Psychology Research Ethics Committee (PRE.2021.092).

## Conflicts of Interest

The authors declare no conflicts of interest.

## Supporting information

Supporting information.

## Data Availability

The data that support the findings of this study are available via the University of Cambridge Data Repository (https://doi.org/10.17863/CAM.107954). Formal requests to PCU_DATA@medschl.cam.ac.uk for access to pseudo‐anonymised transcripts will be considered via a data‐sharing agreement that indicates the criteria for data access and conditions for research use and will incorporate privacy and confidentiality standards to ensure data security. The study protocol, survey and topic guide are available on the repository. Data will be available upon publication with no end date as indicated.
