# Supplementary material for: Understanding the Preferences and Considerations of the Public Towards Risk‐Stratified Screening for Colorectal Cancer: Insights From Think‐Aloud Interviews Based on a Discrete Choice Experiment
Source: Health Expect. 2024 Jul 19;27(4):e14153. doi: 10.1111/hex.14153 (PMC11258464; doi:10.1111/hex.14153)
Supplement: Supplementary file 1 — Supporting information. [file HEX-27-e14153-s001.pdf]

Understanding the preferences and considerations of the public towards risk-stratified screening for colorectal cancer: Insights from think-aloud interviews based on a discrete choice experiment

Supplementary material

Supplementary Table 1. Additional participant characteristics.

	N (%)
Total *N*	20 (100)
Gender identity	
Same as sex assigned at birth	20 (100)
Region of England	
London	8 (40)
South-east	4 (20)
Yorkshire	4 (20)
Other	4 (20)
Self-reported weight	
About the right weight	11 (55)
Slightly or very overweight	9 (45)
Familiarity with cancer through a close friend	
Yes	9 (45)
No	11 (55)

Supplementary Table 2. Participants' thoughts and beliefs about cancer and screening.

	N (%)
Total *N*	20 (100)
**Thoughts and beliefs about cancer**	
"These days, many people with cancer can expect to continue with normal activities and responsibilities"	
Strongly agree	1 (5)
Agree	12 (60)
Neither disagree nor agree	6 (30)
Disagree	1 (5)
Strongly disagree	0 (0)
"Most cancer treatment is worse than the cancer itself"	
Strongly agree	2 (10)
Agree	4 (20)
Neither disagree nor agree	9 (45)
Disagree	5 (25)
Strongly disagree	0 (0)
"I would not want to know if I have cancer"	
Strongly agree	0 (0)
Agree	0 (0)
Neither disagree nor agree	0 (0)
Disagree	5 (25)
Strongly disagree	15 (75)
"Cancer can often be cured"	
Strongly agree	4 (20)
Agree	12 (60)
Neither disagree nor agree	4 (20)
Disagree	0 (0)
Strongly disagree	0 (0)
"Going to the doctor as quickly as possible after noticing a symptom of cancer could increase the chances of surviving"	
Strongly agree	18 (90)
Agree	2 (10)
Neither disagree nor agree	0 (0)
Disagree	0 (0)
Strongly disagree	0 (0)
"Some people think a diagnosis of cancer is a death sentence. To what extent do you agree or disagree with this?"	
Strongly agree	5 (25)
Agree	13 (65)
Neither disagree nor agree	1 (5)
Disagree	0 (0)
Strongly disagree	1 (5)
"How likely do you think it is that you will get cancer at some point in the next 10 years"	
Extremely or moderately likely	2 (10)
Slightly likely	3 (15)

Neither likely nor unlikely	12 (60)
Slightly unlikely	1 (5)
Extremely or moderately unlikely	2 (10)
During the past month:<br>"How often have you thought about your own chances of getting cancer?"	
Not at all	7 (35)
Rarely	6 (30)
Sometimes	5 (25)
Often	2 (10)
A lot	0 (0)
"How often have thoughts about your chances of getting cancer affected your mood?"	
Not at all	10 (50)
Rarely	2 (10)
Sometimes	5 (25)
Often	2 (10)
A lot	1 (5)
"How often have thoughts about your chances of getting cancer affected your ability to perform your daily activities?"	
Not at all	13 (65)
Rarely	4 (20)
Sometimes	3 (15)
Often	0 (0)
A lot	0 (0)
Thoughts and beliefs about cancer screening	
"Do you think that benefits of cancer screening outweigh the possible side effects, potential harms and inconvenience?"	
Yes, for everyone	11 (55)
No, for everyone	0 (0)
It depends on your age and sex	5 (25)
It depends on the type of cancer	6 (30)
It depends on how you feel about caner	2 (10)
It depends on how you feel about screening tests	3 (15)
